# *Granulovirus* GP37 Facilitated ODVs Cross Insect Peritrophic Membranes and Fuse with Epithelia

**DOI:** 10.3390/toxins11030145

**Published:** 2019-03-04

**Authors:** Xiangyang Liu, Wei Fang, Rui Fan, Linna Zhang, Chengfeng Lei, Jingjing Zhang, Wenkai Nian, Tao Dou, Shiheng An, Lin Zhou, Xiulian Sun

**Affiliations:** 1 College of Plant Protection, Henan Agricultural University, Zhengzhou 450002, China; fangwei635@126.com (W.F.); fanrui93@126.com (R.F.); hnndlxy803@163.com (L.Z.); zhangjj@henau.edu.cn (J.Z.); nianwenkai1020@126.com (W.N.); doutao2018@126.com (T.D.); shihengan@hotmail.com (S.A.); zhoulin@henau.edu.cn (L.Z.); 2 Wuhan Institute of Virology, Chinese Academy of Sciences, Wuhan 430071, China; cflei@wh.iov.cn (C.L.); sunxl@wh.iov.cn (X.S.)

**Keywords:** *Cydia pomonella granulovirus*, GP37, peritrophic membrane, brush-border membrane vesicle, synergistic mechanism

## Abstract

The *Cydia pomonella granulovirus* (CpGV) GP37 has synergistic effects on the infectivity of *nucleopolyhedroviruses* (NPVs), however, the mechanism employed is unclear. In this study, in vitro and in vivo binding assays indicated that GP37 efficiently bound to the midgut peritrophic membrane (PM) of *Spodoptera exigua* larvae. Treatment with GP37 led to the damage of the PM’s compacted structure and the generation of the PM perforations, and the enhancement of the PM’s permeability. qPCR results further demonstrated that GP37 increased the ability of occlusion-derived virions (ODV) to cross the PM. R18-labeling experiments exhibited that GP37 also promoted the fusion of ODVs and insect midgut epithelia. Altogether, our present results revealed that the synergistic mechanism of GP37 to the infectivity of NPV might involve two parts. GP37 damaged the integrity of the PM after binding, which enhanced the PM’s permeability and increased the ability of ODVs to cross the PM, finally facilitating the ODVs reaching the midgut. In addition, GP37 promoted the fusion of ODVs and insect midgut epithelia. Our data expand the understanding of the mechanism used by baculovirus synergistic factors and provide a foundation for the development of high-efficiency baculoviral insecticides.

## 1. Introduction

Baculoviridae are pathogenic for insects and have large double-stranded DNA genomes of 80–180 kb [1] and produce two forms of virions; an occluded form that is present in the occlusion bodies, and a budded form that spreads the infection within the insect [2]. After ingestion by insect larvae, occlusion bodies are dissolved in the insect midgut releasing the virions. The midgut of the insect is lined with a structure called the peritrophic matrix (PM) that is composed of proteins and chitin [3] and serves as a barrier to protect the midgut cells from damage from the vegetation that the larvae consume and from pathogens. In order to initiate an infection, virions must pass through the PM to access the midgut cells. A glycoprotein that might facilitate this access is called GP37. It is encoded by most Alphabaculoviruses and a few Betabaculoviruses [1]. A related protein with 30–40% amino acid sequence identity called fusolin is encoded by entomopoxviruses. Both GP37s and fusolins contain a predicted chitin-binding domain [4,5,6]. Fusolins accumulate in bi-pyramidal spindle-shaped occlusion bodies and, similarly, GP37 is also present in occlusion bodies [7,8,9,10] and is associated with both occlusion-derived virions (ODV) and budded viruses [11].

It has been reported that entomopoxvirus fusolin plays a role in the enhancement of entomopoxvirus infection [12,13] by causing disruption of the PM [14]. Structural analysis of entomopoxvirus spindles indicated that they contained a globular domain that is related to lytic polysaccharide monooxygenases of chitinovorous bacteria. It is thought that upon ingestion by the host, the spindles are dissolved, and the monooxygenase domain is exposed and can then digest the chitin-rich PM [15]. Similar to fusolin, the GP37 of *Cydia pomonella granulovirus* (CpGV) was found to bind chitin and was able to enhance per os infections [16,17]. In this report, experiments examining the role of GP37 of CpGV in causing in disruption of the PM and facilitating the viral infection are described.

## 2. Results

### 2.1. Expression of CpGV GP37

**Figure 1:**
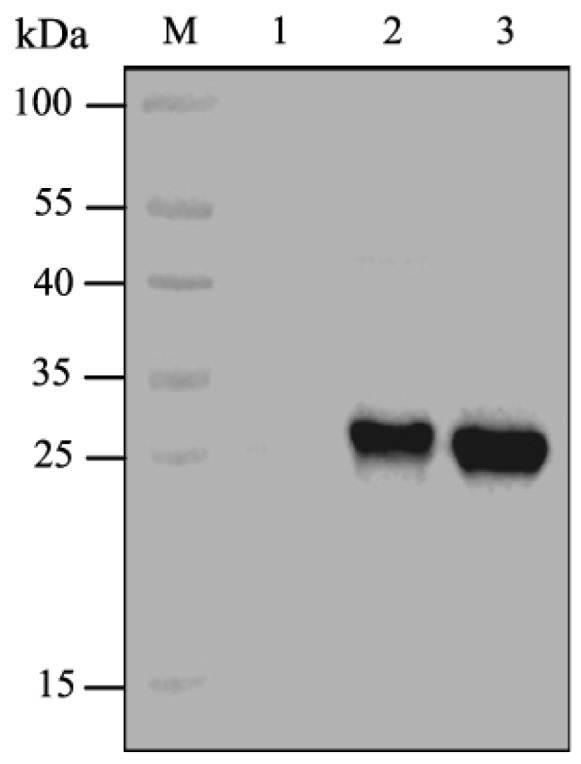
Western blot analyses of cell-free expression of complete and a truncated *Cydia pomonella granulovirus* (CpGV) GP37. The 756-bp complete fragment and truncated 663-bp segment of CpGV *gp37* were amplified. Complete and truncated GP37s were expressed using the TNT T7 insect cell extract protein expression system, and expression products were separated by sodium dodecyl sulfate polyacrylamide gel electrophoresis (SDS-PAGE) and then detected using western blot. M, protein marker; lane 1, negative control; lane 2, complete GP37; lane 3, GP37 (del 1–93 nt).

Complete and truncated CpGV GP37s were expressed using the TNT T7 insect cell extract protein expression system (Promega, Madison, WI, USA). Results demonstrated that the molecular weight of the expressed GP37 protein (full-length) was ~30 kDa (Figure 1, lane 2), consistent with the molecular weight of the predicted GP37. The molecular weight of the expressed GP37-truncated protein (del 1–31 aa) was ~27 kDa (Figure 1, lane 3), which was in accordance with the predicted molecular weight plus the fused tag of the pF25A vector. Liu et al. [15] had identified that the CpGV GP37 (del 1–31 aa) showed synergistic effects on *nucleopolyhedroviruses* (*NPVs*), thus we used the truncated version of GP37 in this study.

### 2.2. Binding of GP37 to PM

**Figure 2:**
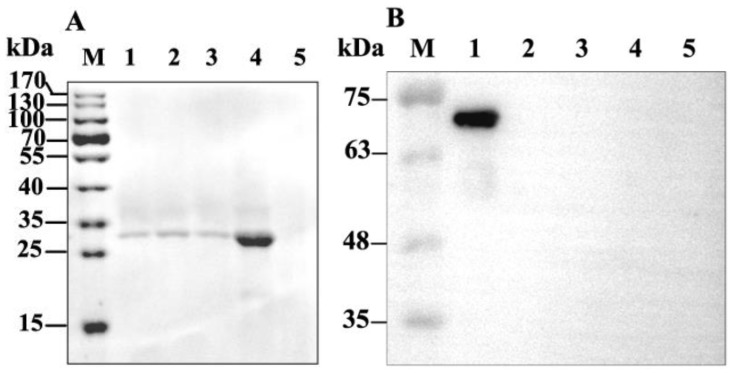
Immunobloting analysis of GP37 (**A**) and bovine serum albumin (BSA) (**B**) binding to the peritrophic membrane (PM) of *S. exigua* larvae in vitro. PMs of healthy *S. exigua* larvae were incubated in vitro with CpGV GP37 or BSA for 1, 3 and 6 h. After incubation, PMs were separated by SDS-PAGE and analyzed by western blot. (**A**): M, protein marker; lane 1, 2 and 3, PM incubated with GP37 1 h, 3 h, and 6 h; lane 4, CpGV GP37 expressed in cell free system; lane 5, PM incubated with distilled water 6 h (control). (**B**): M, protein marker; lane 1, BSA; lane 2, 3 and 4, PM incubated with BSA 1 h, 3 h, and 6 h; lane 5, PM incubated with distilled water 6 h (control).

When the PMs were isolated from the *S. exigua* larvae, western blot results demonstrated that a 27-kDa band was detected in the PMs incubated with GP37 for 1 h, 3 h and 6 h (Figure 2A). In contrast, no immunoband was detected in the PMs incubated with bovine serum albumin (BSA) (Figure 2B). In addition, no immunoband was detected in the control (Figure 2), suggesting that GP37 was capable of specifically binding with PMs in vitro.

**Figure 3:**
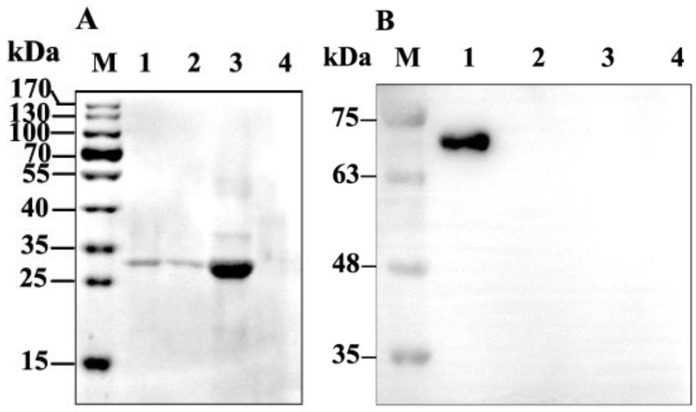
Immunobloting analysis of GP37 (**A**) and BSA (**B**) binding to the PM of *S. exigua* larvae in vivo. Fifth-instar *S. exigua* larvae were fed with CpGV GP37 or BSA and then dissected after 3 and 6 h of feeding. PMs were harvested and analyzed by western blotting after separation by SDS-PAGE. (**A**): M, protein marker; lane 1 and 2, PM of *S. exigua* larvae after feeding GP37 for 3 h and 6 h; lane 3, CpGV GP37 expressed in cell free system; lane 4, PM of healthy *S. exigua* larvae fed with artificial diet (control). (**B**): M, protein marker; lane 1, BSA; lane 2 and 3, PM of *S. exigua* larvae after feeding BSA for 3 h and 6 h; lane 4, PM of healthy *S. exigua* larvae fed with artificial diet (control).

When GP37 was fed to *S. exigua* larvae for 3 h and 6 h, western blot analysis also showed that the same 27 kDa GP37 was detected in all PM samples (Figure 3A). However, this band was not present in PMs from artificial diet-fed larvae (without GP37 and BSA) (Figure 3A) and BSA-fed larvae (Figure 3B), indicating that GP37 bound to the PM specifically in vivo.

### 2.3. GP37 Caused Perforations on PMs

**Figure 4:**
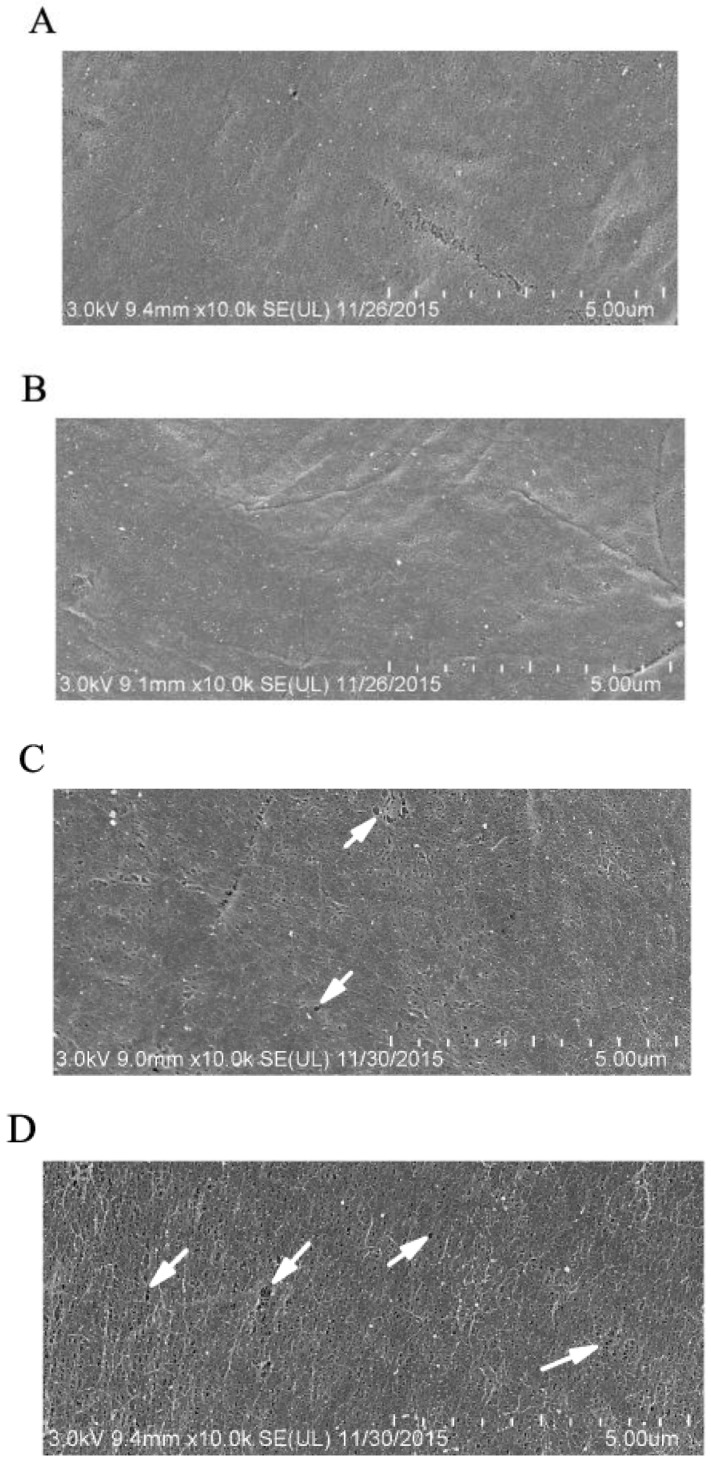
Scanning electron micrographs of *S. exigua* larvae PM. *S. exigua* larvae were dissected after feeding GP37 for 3 and 7 h, and the PMs were harvested. The morphological characteristics of PMs were observed using SEM. (**A**) and (**B**) PM of *S. exigua* larvae after feeding artificial diet for 3 h and 7 h, respectively. (**C**) and (**D**) PM of *S. exigua* larvae after feeding GP37 for 3 h and 7 h, respectively. White arrows indicate perforations caused by GP37.

Compared to healthy *S. exigua* PMs with a smooth compacted morphology (Figure 4A,B), feeding with GP37 for 3 h led to a significant change in PM structures, which no longer appeared smooth and compact, and small perforations were observed (Figure 4C). Further feeding for a longer time (7 h) led to more and larger perforations on the PM (Figure 4D). SEM observations revealed that CpGV GP37 damaged the smooth compacted structure of the PM and led to the appearance of perforations.

### 2.4. GP37 Enhanced PM Permeability

To analyze the effects of GP37 on the permeability of insect PMs, the fluorescence of the middle piece of the midgut was observed using an inverted fluorescence microscope. Midguts from larvae fed with only distilled water or GP37 showed no fluorescence (Figure S1A,B), weak fluorescent signals were detected in the midguts of *S. exigua* fed with distilled water + FITC-dextran 70 kDa or distilled water + FITC-dextran 500 kDa (Figure S1C,D), and strong fluorescent signals were detected in the midguts of *S. exigua* fed with FITC-dextran 70 kDa + GP37 or FITC-dextran 500 kDa + GP37 (Figure S1E,F).

**Figure 5:**
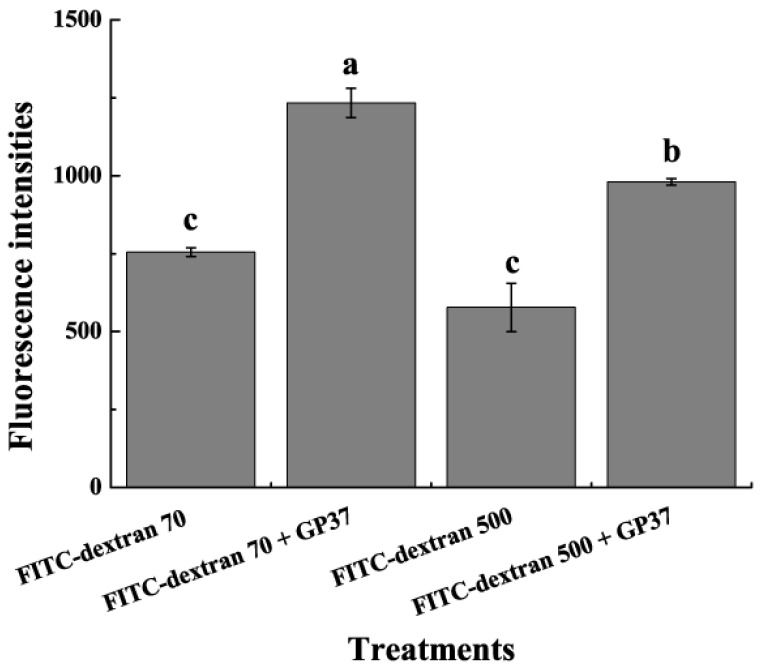
The fluorescence intensity levels of midguts and intestinal juices from *S. exigua* larvae fed with FITC-dextran supplemented with or without GP37. Four groups of uniform-sized late fourth-instar *S. exigua* larvae were fed with FITC-dextran 70 kDa, FITC-dextran 500 kDa, GP37 + FITC-dextran 70 kDa, and GP37 + FITC-dextran 500 kDa, respectively. *S. exigua* larvae were dissected after 2 h, and the midguts and intestinal juices were collected. The fluorescence intensities of midguts and intestinal juices were determined using a microplate reader. Data show the mean ± SE for three repetitions. Different letters above bars indicate significant differences among treatments (separated by Duncan’s new multiple range test after one-way ANOVA, *P* < 0.05).

To analyze the fluorescence of particles that passed through the PM precisely, the fluorescence intensities of midguts and intestinal juices were determined using a microplate reader. The fluorescence intensity level of the combined midguts and intestinal juices of *S. exigua* treated with FITC-dextran 70 kDa + GP37 was 1,233.67 ± 46.80, which was significantly greater than that of the FITC-dextran 70 kDa-treated larvae (754.33 ± 14.00, *P* < 0.05) (Figure 5). The fluorescence intensity level of the combined midguts and intestinal juices of *S. exigua* treated with FITC-dextran 500 kDa + GP37 was 980.00 ± 10.35, which was significantly greater than that of the FITC-dextran 500 kDa-treated larvae (577.33 ± 77.90, *P* < 0.05) (Figure 5). Thus, exposure to GP37 increased the amount of FITC-dextran 70 kDa and 500 kDa that passed through the PM.

### 2.5. AcMNPV DNA Contents in Midguts and Intestinal Juices

To investigate the effect of GP37 on the number of viral particles crossing PMs, the *Ac-odv-e25* copy number in *S. exigua* larvae fed with *Autographa californica multiple NPV* (AcMNPV) with or without the presence of GP37 was checked. Results demonstrated that the *Ac-odv-e25* copy number was 5.07 (±0.32) × 10^7^ copies/µL in *S. exigua* larvae fed with AcMNPV + GP37, which was significantly more than that in *S. exigua* larvae fed with AcMNPV [0.41 (± 0.13) × 10^7^ copies/µL] (*t* = 13.478, *P* < 0.01). These results indicated that GP37 increased the amount of ODV that passed through the PM and reached the midgut.

### 2.6. GP37 Facilitated the Fusion of ODVs and Midgut Epithelia

The ODVs labeled with octadecyl rhodamine B chloride (R18) (ODVRs) were used for fusion experiments with brush-border membrane vesicles (BBMVs), and the effects of GP37 on their ability to fuse were analyzed by determining the changes of BBMV fluorescence intensity levels when adding different GP37 concentrations (Table 1). The fluorescence intensity was 0.0814 when 2 µg/mL of GP37 was added, which was not significantly different from when no GP37 was added (BBMV + ODVR; 0.0858). When 6 µg/mL of GP37 was added, the measured fluorescence intensity increased to 0.1123, which was significantly greater than 0.0777 in the BBMV + ODVR-treated group (*P* < 0.05). The fluorescence increased to 0.1277 when 10 µg/mL of GP37 was added, and the fluorescence intensity level was significantly greater than that in the BBMV + ODVR-treated group, which was 0.0749 (*P* < 0.05). Thus, GP37 showed promotive effects on the fusion of ODVs and midgut epithelia with a dose-dependent pattern.

## 3. Discussion

The baculovirus infection process begins in the insect’s midgut. The viral external polyhedron protein of occlusion bodies (OBs) is hydrolyzed in the alkaline environment of the midgut (pH 8–11), resulting in the release of the ODVs. Released ODVs cross the PM and then infect the midgut epithelial cells by fusing with the microvilli of the midgut, in which virus genes are transcribed, replicated and assembled and the initial infection is completed. Meanwhile, ODVs that fail to pass through the PM are passed out of the body in the feces [1]. The PM is an invertebrate-specific midgut endomembrane-like tissue that mainly consists of chitins and proteins. Approximately 10 chitin microfibrils develop in parallel to form microfibril bundles, and these bundles are further arranged into a dense mesh framework by intersecting at certain angles. This chitin mesh framework is tightly bound by peritrophins, including intestinal-type mucins and chitin-binding proteins, through their chitin-binding domains. Gaps in the framework are filled with polysaccharides, which together form a semi-permeable membrane [3]. The pore diameters of PMs are typically in the range of 21–29 nm in lepidopteran insects and 24–36 nm in orthopteran insects [17]. The PM is not only a biochemical barrier, insulating or even inactivating toxins that enter the insect, but also a physical barrier, protecting the midgut epithelial cells from damage caused by food particles and digestive enzymes, and, in particular, from infection by pathogens, such as baculoviruses [3,18]. Therefore, the PM has critical effects on the baculovirus infection process.

Liu et al. [15] reported that the synergistic effects of CpGV GP37 significantly enhanced the insecticidal activities of *Bacillus thuringiensis* and *NPV*. However, the detailed mechanism remained elusive. GP37 contains a chitin-binding domain, allowing in vitro binding to free chitins [15]. Here, our results revealed that CpGV GP37 could bind to *S. exigua’*s PM, which conformed to the PM-binding assay of its homologue *Spodoptera litura multiple NPV* GP37 [11]. GP37 should bind to PMs on the chitin framework because it contains a chitin-binding domain. Thus, the binding of GP37 to the chitin framework of the PM, instead of to peritrophins, might affect its compactness or even its integrity. The binding of PM and GP37 inspired us to further investigate the effect of binding on PM. The PM’s morphology was observed using SEM, and a normal PM was smooth and compacted, while the morphology changed, having greater pore diameters and even perforations, after 3–7 h of GP37 feeding (Figure 4). The SEM observations indicated that GP37 damaged the smooth, compacted PM structure and caused perforations.

The appearance of perforations with greater diameters may indicate that PM permeability increased and made the corresponding blocking capability weaker. To measure the effects of GP37 on the insect PM’s permeability, FITC-dextran 70 kDa and 500 kDa were used as indicators, and midgut fluorescence was observed using an inverted fluorescence microscope. A stronger fluorescence was detected in the GP37-treated group. On the basis of this result, the fluorescence intensity levels of midguts and intestinal juices were measured using a microplate reader. The results corroborated the inverted fluorescence microscope observations, in which stronger fluorescence intensities were observed in the midguts and intestinal juices when GP37 was present. Thus, GP37 may damage the compactness and integrity of the PM through direct interactions with the PM. The interactions increased the PM’s permeability and decreased its blocking ability. These results are also consistent with qPCR results, which confirmed that the *Ac-odv-e25* gene’s copy number in the midgut and intestinal juice increased significantly in the presence of GP37. These results altogether revealed that more ODV crossed the PM and reached the midgut after the increase in the PM’s permeability and the decrease in its blocking capability. Generally, the diameters of ODVs released from the OBs of baculoviruses, such as *Epinotia granitalis NPV* [19], *Iragoidae fasciata NPV* [20] and *Epinotia aporema GV* [21], are greater than 40 nm. Thus, the ODV’s diameters are greater than the normal PM’s perforation diameter. Therefore, factors that facilitate the passage of virions through the PM toward the midgut epithelium are necessary, and GP37 is this kind of enhancing factor.

ODVs attachment to and fusion with midgut epithelial cells are critical steps in baculovirus infection and BBMVs act as a bridge between ODVs and midgut epithelial cells. Horton and Burand [22] have pointed out that ODV could fuse with BBMVs and then release virions into the midgut epithelial cells to initiate primary infection. The activity levels of aminopeptidases reflected the quality of BBMVs. In this study, the high activity levels of aminopeptidases in the prepared BBMVs were indicated by measuring the production of 4-nitroaniline using L-leucine-*p*-nitroanilide as the substrate (data not shown). Thus, BBMVs could be used for fusion experiments. By determining the fluorescence intensity level after the fusion of BBMVs and ODVR, we found that GP37 promoted the fusion of ODVs and BBMVs. A previous report showed that the fusion of *Pseudaletia unipuncta nucleopolyhedrovirus* and insect cells was enhanced by fusolin, a GP37 homologue [23], so it was credible that GP37 promoted the fusion of ODVs with insect midgut epithelia. However, this promotive effect was restricted to certain GP37 levels. Levels less than 2 µg/mL showed no significant promotive effects. Liu et al. [15] reported the significant synergistic effect of GP37 when feeding insects 30 µg/mL of GP37. Based on our results, if all 30 µg/mL of GP37 acted on ODV and BBMV fusion, then no significant synergistic effects should have been detected. Thus, we proposed that, when GP37 was swallowed by insects, it first bound with PM chitins, then damaged the normal PM structures and finally caused perforations. In addition, the remaining GP37 passed through the damaged PM together with ODV and reached the midgut, where GP37 promoted the fusion of ODVs and BBMVs (epithelia).

It has been reported that the effects of some baculoviral synergistic factors are related to the insect’s PM. Factors such as chitinase [24], calcofluor [25] and chlorfluazuron [26] damage the PM’s integrity by interacting with chitins, while enhancin [27,28,29] and ScathL [30] increase the PM’s permeability by degrading PM proteins. Additionally, fusolin causes PM perforations using its unique structure [7,14]. Although the synergistic effects of baculoviral GP37 have been reported [15], its synergistic mechanism remained unknown. Here, GP37 was shown to interfere with the normal formation of PMs through direct interactions, resulting in higher PM permeability. In addition, GP37 promoted the fusion of ODVs and BBMVs. On the basis of these results, we hypothesized that GP37 acts at two critical points of the baculoviral infection process. The first critical point is when the ODVs cross the PM. After being swallowed by insects, the baculoviral polyhedron proteins are digested, and ODVs are released. The insect becomes infected by the virus if the ODVs cross the PM. The infections’ severity level depends on the number of ODVs that cross the PM. GP37 enhanced the PM’s permeability through direct interactions, allowing more ODV to pass through the PM and reach the midgut. The second critical point was the fusion of ODVs and BBMVs. After the PM’s permeability was increased by GP37, not only ODVs but also GP37 itself, passed through the PM easier. When the integrity of the PM was damaged, the remaining GP37 crossed the PM together with ODVs, reached the midgut, and then induced the fusion of ODVs and BBMVs.

In summary, the synergistic effects of GP37 have been shown, but its synergistic mechanism remained unclear. In this study, using the successful cell-free expression of GP37, the binding of GP37 with insect PMs and the effects of GP37 on PM formation and structures were studied, indicating that GP37 damaged the normal PM’s structure, increasing its permeability, which allowed the ODVs to pass through the PM and reach the midgut easier. The increased PM permeability benefited not only ODVs but also GP37 itself. The remaining GP37 passed through the PM together with ODVs and reached the midgut epithelial cells. Then, GP37 promoted the fusion of ODVs and BBMVs. This study presents a theoretical mechanism for baculovirus synergistic factors, providing a foundation for the development of targeted high-efficiency baculoviral insecticides and indicating possible solutions for preventing or overcoming the resistance of host insects to baculoviruses.

## 4. Materials and Methods

### 4.1. Insects and Viruses

*S. exigua* larvae were obtained from the Core Facility Center and Technical Support, Wuhan Institute of Virology, Chinese Academy of Sciences. The larvae were reared on a bean-flour-based artificial diet [31] and maintained in the laboratory at 27 ± 1 °C with 70–80% relative humidity under a light:dark regime of 14 h:10 h. AcMNPV and CpGV were kindly provided by Dr. Xiulian Sun of Wuhan Institute of Virology, Chinese Academy of Sciences. These two viruses were propagated by infecting late second-instar larvae and preserved in our laboratory.

### 4.2. Expression of CpGV GP37

The CpGV genomic DNA was extracted from OBs according to the method of Smith and Crook [32]. Three primers were designed based on the *gp37* sequence of CpGV. To purify the expressed GP37, the His-tag sequences were inserted into reverse primer gp-r (Table 2). Primers were synthesized by Sangon Biotech Co., Ltd. (Shanghai, China).

The full-length *gp37* was amplified using the CpGV genome as the template and primers (gp-f1 and gp-r). A truncated 663-bp segment of *gp37* (without signal sequences and transmembrane helices at the N-terminus) was amplified by gp-f2 (located 93-nt downstream of the *gp37* ATG translational initiation codon) and gp-r. Two PCR products were ligated into the pMD19-T vector, named as pMD19-T-*gp37* and pMD19-T-*gp37*(del 1–93 nt), respectively. pMD19-T-*gp37* and pMD19-T-*gp37*(del 1–93 nt) were digested with *Sgf*I and *Pme*I, and the resulting fragments were cloned into the pF25A T7 flexi vector (Promega, Madison, WI, USA). The resulting recombinant plasmids were named pF25A-*gp37* and pF25A-*gp37*(del 1−93 nt), respectively.

CpGV GP37 was expressed using the TNT T7 insect cell extract protein expression system according to the manufacturer’s instructions. Plasmids (4 µg) pF25A-*gp37* and pF25A-*gp37*(del 1−93 nt) were independently added to the system, which contained 40 µL of TNT T7 ICE master mix (Promega, Madison, WI, USA), and the total volume was set as 50 µL. The mixtures were then incubated at 28 °C for 4 h. The expressed proteins were detected by western blots using a CpGV GP37 polyclonal antibody (prepared previously by Liu et al.) [15] as the primary antibody and an alkaline phosphatase-labeled goat anti-rabbit IgG antibody as the secondary antibody. Expressed GP37 was purified with Ni-NTA His•Bind^®^ Resins, according to the manufacturer’s protocol (Novagen, Madison, WI, USA).

### 4.3. In Vitro PM-Binding Experiment

Healthy fifth-instar *S. exigua* larvae were dissected according to Derksen and Granados [33] with modifications. Briefly, fifth-instar *S. exigua* larvae were paralyzed by incubating on ice for 10 min and then transferred into phosphate-buffered saline (PBS; pH 7.4). Epidermides were lacerated along the midline using tweezers, and the midgut was exposed. The midgut was lacerated using the same method, and the PM was carefully separated and washed three times with deionized water. Isolated PMs were placed into 1.5-mL centrifuge tubes, with 10 PMs per tube. PMs were in vitro incubated respectively with CpGV GP37 (20 µg/mL) and BSA (for negative control) (20 µg/mL) at 27 °C for 1, 3 and 6 h and then washed three times using distilled water, air dried, ground in a mortar and then transferred into new 1.5-mL centrifuge tubes. In addition, PMs incubated with distilled water were used as a control. After adding 100 µL PBS buffer, tubes were centrifuged at 1200× *g* for 15 min, and the supernatants were collected and then analyzed by western blot using GP37 polyclonal antibody and rabbit anti-bovine serum albumin polyclonal antibody (Bioss, Woburn, MA, USA) as the primary antibody, respectively. An alkaline phosphatase-labeled goat anti-rabbit IgG antibody was used as the secondary antibody.

### 4.4. In Vivo PM-Binding Experiment

In total, 60 fifth-instar *S. exigua* larvae were selected and divided evenly into two groups; one group was fed with BSA (20 µg/mL), and the other group was fed with the GP37 protein (20 µg/mL). The larvae fed with artificial diet (without GP37 and BSA) were used as controls. *S. exigua* PMs were harvested after 3 and 6 h of feeding using the method described above. Isolated PMs were ground in a mortar and placed in 100 µL PBS. The supernatant was collected after centrifugation at 1200× *g* for 15 min and analyzed by western blot using the method described above.

### 4.5. Scanning Electron Microscope (SEM) Observations of PM Structures

Uniform-sized, healthy fifth-instar *S. exigua* larvae were selected, fed with CpGV GP37 (50 µg/mL) after an 8-h starvation treatment. PMs were harvested from *S. exigua* midguts at 3 and 7 h post feeding. PMs isolated were then treated using the method reported by Mitsuhashi et al. [7] and further observed using SEM (Hitachi, Tokyo, Japan). Healthy *S. exigua* larvae fed with artificial diet (without GP37) were used as controls.

### 4.6. Determination of PM Permeability

Uniform-sized fifth-instar *S. exigua* larvae were selected and divided into six groups named A, B, C, D, E, and F. After 8 h of starvation, *S. exigua* larvae were fed with different treatments using the droplet-feeding method [34]. Group A received only distilled water, group B received 50 µg/mL GP37, group C received distilled water + 20 mg/mL fluorescein isothiocyanate dextran (FITC-dextran) 70 kDa, group D received distilled water + 20 mg/mL FITC-dextran 500 kDa, group E received 50 µg/mL GP37 + 20 mg/mL FITC-dextran 70 kDa, and group F received 50 µg/mL GP37 + 20 mg/mL FITC-dextran 500 kDa. Each group contained 10 larvae, and four replicates were employed per group. The artificial diets were added immediately after droplets were swallowed by larvae. Midguts and intestinal juices were collected 2 h after treatment using a method reported by Mitsuhashi et al. [7].

One midgut was selected randomly and cut into three pieces, and the fluorescence of the middle piece was observed using an inverted fluorescence microscope (Olympus, Tokyo, Japan). The remaining midguts and intestinal juices were ground in a mortar and centrifuged for 30 min at 3000× *g*. Aliquots of supernatants were supplemented with PBS to a total volume of 200 µL and mixed slowly on a horizontal shaker for 30 min. Then, the fluorescence intensities were measured with a microplate reader (Bio-Tek, Winooski, VT, USA) using a 485-nm excitation light and emitted light was monitored at 520 nm. The whole process was performed in the absence of light. The results were analyzed using a one-way analysis of variance in SPSS 19.0 (SPSS Inc., Chicago, IL, USA) after arcsine square-root transformation. After the significant differences in the overall means were identified using F-tests, the averages of each pair of groups were compared using Duncan’s new multiple range test.

### 4.7. Real-Time qPCR Qualification of AcMNPV DNA Levels

AcMNPV DNA levels in midguts and intestinal juices were detected by real-time qPCR. In total, 200 fifth-instar *S. exigua* larvae were starved for 8 h and divided evenly into two groups that were fed with 1.0 × 10^8^ OBs/mL AcMNPV and 1.0 × 10^8^ OBs/mL AcMNPV+ 50 µg/mL GP37, respectively. Larvae were transferred to the ice after 6 h, dissected one by one after paralysis, and the midgut juice and midgut epithelial cells between the PM and midgut were collected.

The conserved gene *Ac-odv-e25* in AcMNPV was selected as a marker to detect virus quantity. The primers odv-e25f (ATGTGGGGAATCGTGTTACT) and odv-e25r (CGATGCGCAAGTTGGCCAC) were designed and synthesized.

Collected midguts and intestinal juices were ground in a mortar together with 200 µL of PBS buffer. Viral particle DNA was extracted from 200 µL of ground fluid using a viral DNA kit (Omega Bio-Tek, Norcross, GA, USA). Using the DNA as template, real-time qPCR was performed to determine the viral quantity in insect intestinal juices and midgut epithelial cells using the primer pair odv-e25f and odv-e25r. Two-step qPCR was performed. Step 1 was 95 °C for 30 s, and step 2 was 40 cycles of 95 °C for 5 s and 60 °C for 30 s. A melt curve was then performed. Data from three replicates were pooled to calculate the viral quantity as long as there was no significant difference between the results of the three replicates. The quantity differences in viral particles passing through AcMNPV- and AcMNPV+GP37-treated larval PMs were compared by t-test using SPSS19.0 (SPSS Inc., Chicago, IL, USA).

### 4.8. Extraction of BBMVs from S. exigua Midguts

The extraction of BBMVs was performed using the method of Wolfersberger et al. [35] with slight modifications. Midguts were isolated from healthy fifth-instar *S. exigua* larvae and mixed with MET buffer [300 mM mannitol, 5 mM ethylenebis (oxyethylenenitrilo) tetraacetic acid and 20 mM Tris-HCl (pH 7.4)] at a 1:9 (w/v) ratio, ground in an ice-chilled mortar with 1 mM phenylmethanesulfonyl fluoride. Then, an aliquot of 24 mM MgCl_2_ was added and mixed. The samples were incubated on ice for 30 min, and the supernatants were collected after centrifugation at 1200× *g* for 15 min at 4 °C. The supernatants were centrifuged at 17,000× *g* for 2 h at 4 °C, and the resulting pellets were BBMVs of midgut epithelia. The BBMV pellets were re-suspended in the same volume of MET-MgCl_2_ buffer and were purified by three rounds of differential centrifugation at 2500× *g* for 15 min and then one round at 17,000× *g* for 2 h. The final BBMV pellets were suspended using 0.5× MET buffer and stored in liquid nitrogen. The BBMV protein concentrations were measured using the Bradford method [36].

### 4.9. R18 Labeling of AcMNPV ODVs

Briefly, 3 mL of 1× DAS solution (0.01 M ethylenediaminetetraacetic acid, 0.1 M Na_2_CO_3_ and 0.17 M NaCl; pH 10.8) was added to 10^9^ OBs/mL AcMNPV (6 mL) and incubated for 5 min on a shaker. The reaction was terminated by adding 1 mL of 1 M Tris-HCl (pH 7.6), and after a 20-min centrifugation at 2000× *g*, the supernatant was considered as the ODV suspension. Then, 40 µL of R18 ethanol solution was added for every 4 mL ODV suspension for labeling. The samples were placed in the dark at 4 °C for 5 min and then centrifuged using a 25%, 35%, 57% and 63% sucrose gradient for 1.5 h. The ODV-containing band was carefully isolated by pipetting, re-suspended using 0.1× TE buffer (10 mM Tris-HCl and 1 mM ethylenediaminetetraacetic acid) and centrifuged for 1 h at 90,000× *g* in 4 °C. The pellets were re-suspended using PBS (4mL) and preserved in −80 °C. R18-labeled ODVs were marked as ODVRs.

### 4.10. Fusion of ODVs and Midgut Epithelia

Briefly, 96-well microtiter plates were coated by a BBMV-containing PBS solution (20 µg) at 4 °C overnight. Plates were washed three times with washing buffer, 15 min per wash. ODVR (10 µL) and 2, 6 and 10 µg/mL GP37 (10 µL) were independently mixed, and the total volumes were supplemented with PBS to 100 µL. Three replicates were performed for each GP37 concentration. The BBMV only group was the blank control, and the BBMV + ODV group was the negative control. Plates were incubated at room temperature for 1.5 h. Then, they were washed three times, 15 min per wash, with washing buffer. Finally, 200 µL PBS was added to each well, and fluorescence intensities were measured using a 560-nm excitation light and emitted light was monitored at 590 nm.

## Figures and Tables

**Table 1:** Fluorescence intensity levels of BBMV fused with ODVR ^†^.

Treatments	GP37 Concentrations (µg/mL)					
	2		6		10	
	Fluorescence Intensity Levels	Significant Differences	Fluorescence Intensity Levels	Significant Differences	Fluorescence Intensity Levels	Significant Differences
BBMV+ODVR+GP37	0.0814 ± 0.0056	a	0.1123 ± 0.0004	a	0.1277 ± 0.0217	a
BBMV+ODVR	0.0858 ± 0.0061	a	0.0777 ± 0.0063	b	0.0749 ± 0.0121	b
BBMV	0.0065 ± 0.0017	b	0.0028 ± 0.0008	c	0.0036 ± 0.0021	c

^†^ Fluorescence intensity levels are shown as means ± standard errors. Different letters behind a fluorescence intensity level indicate significant differences among treatments (separated by Duncan’s new multiple range test after one-way ANOVA, *P* < 0.05).

**Table 2:** Primers used for cloning CpGV *gp37* gene.

Primer	Primer Sequences (5′ → 3′)
gp-f1	*GATG*GCGATCGCC ATGATGACGATTATGAAAAATCCC *Sgf*I Site underlined
gp-f2	*AGCT*GCGATCGCC ATGCCGTTGGCGAGACAGCG *Sgf*I Site underlined
gp-r	*GTGC*GTTTAAACTTA**GTGATGGTGATGGTGATG**CAAATCACTTTTCGTTTGC *Pme*I Site underlined

Italics sequences: the protective base; Underline sequences: restriction enzyme site; Bold sequences: his-tag.

## Supplementary Materials

The following are available online at https://www.mdpi.com/2072-6651/11/3/145/s1, Figure S1: The fluorescence of midgut from *Spodoptera exigua* larvae swallowing distilled water (**A**), GP37 (**B**), FITC-dextran 70 kDa (**C**), FITC-dextran 500 kDa (**D**), FITC-dextran 70 kDa + GP37 (**E**), FITC-dextran 500 kDa + GP37 (**F**).
