# Supplementary material for: Granulovirus GP37 Facilitated ODVs Cross Insect Peritrophic Membranes and Fuse with Epithelia
Source: Toxins (Basel). 2019 Mar 4;11(3):145. doi: 10.3390/toxins11030145 (PMC6468389; doi:10.3390/toxins11030145)
Supplement: Supplementary file 1 [file toxins-11-00145-s001.zip › toxins-438869-SI.pdf]

Toxins **2019**, *11*, 145; doi:10.3390/toxins11030145

Supplementary Materials: *Granulovirus* GP37 Facilitated ODVs cross Insect Peritrophic Membranes and Fuse with Epithelia

Xiangyang Liu, Wei Fang, Rui Fan, Linna Zhang, Chengfeng Lei, Jingjing Zhang, Wenkai Nian, Tao Dou, Shiheng An, Lin Zhou and Xiulian Sun

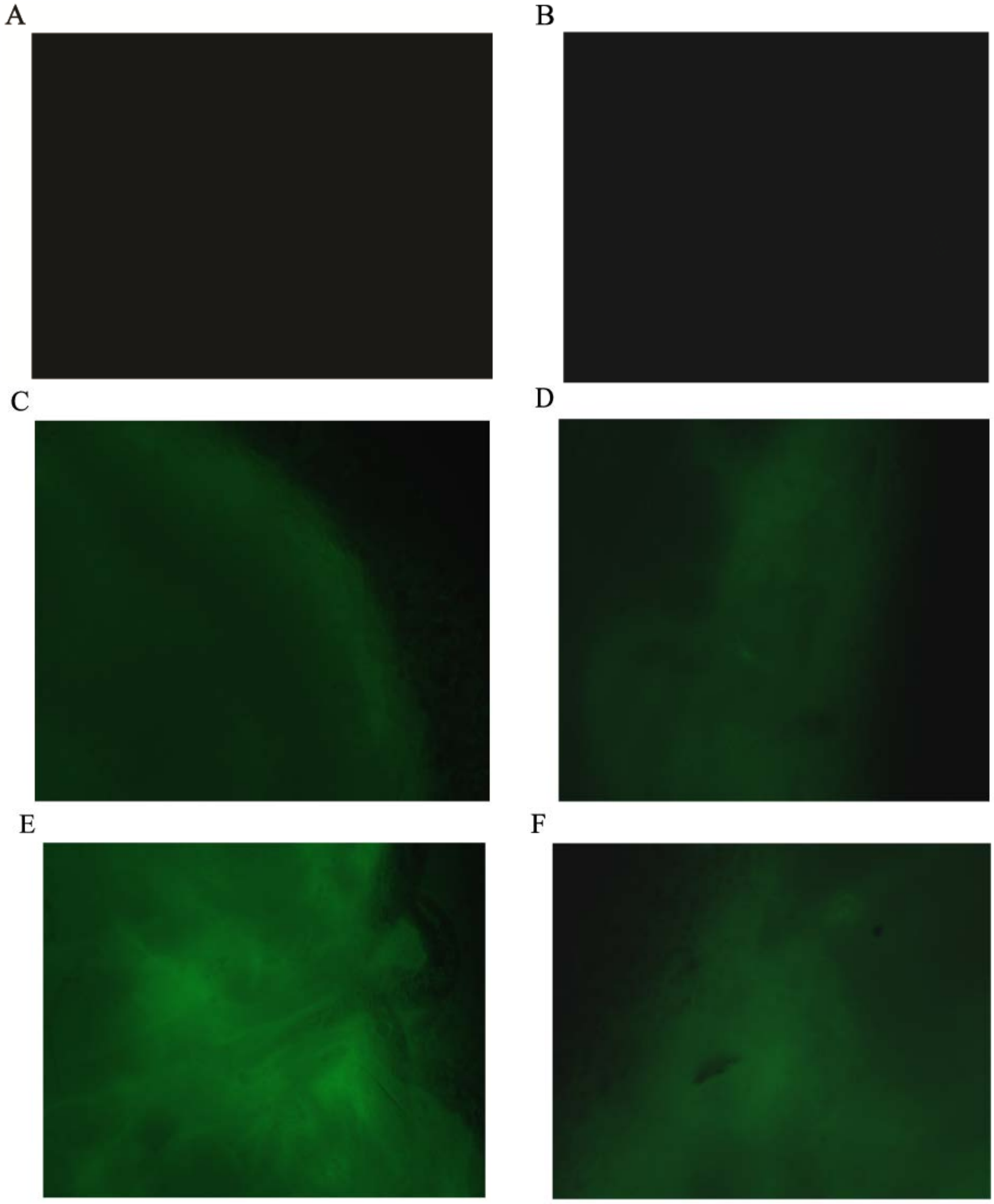

Figure S1. The fluorescence of midgut from *S. exigua* larvae swallowing distilled water (**A**), GP37 (**B**), FITC-dextran 70 kDa (**C**), FITC-dextran 500 kDa (**D**), FITC-dextran 70 kDa + GP37 (**E**), FITC-dextran 500 kDa + GP37 (**F**). Six groups of uniform-sized late fourth-instar *S. exigua* larvae were fed distilled water, GP37, FITC-dextran 70 kDa, FITC-dextran 500 kDa, GP37 + FITC-dextran 70 kDa, and GP37 + FITC-dextran 500 kDa, respectively. *S. exigua* larvae were dissected after 2 h, and the midguts were collected. The fluorescence of the middle piece of the midgut was observed using an inverted fluorescence microscope.
